# Dedifferentiation of Plant Cells: A Term Covering Multiple Pathways?

**DOI:** 10.3390/plants15030479

**Published:** 2026-02-03

**Authors:** Attila Fehér

**Affiliations:** 1 Department of Plant Biology, University of Szeged, 52. Közép Fasor, 6726 Szeged, Hungary; feher.attila@brc.hu or feher.attila.sandor@szte.hu; 2 Institute of Plant Biology, HUN-REN Biological Research Centre, 6726 Szeged, Hungary

**Keywords:** plant cell and tissue culture, protoplast, callus, plant regeneration, cell division, plant hormones, senescence, programmed cell death, signaling

## Abstract

The remarkable plasticity of plants is best exemplified by the capacity of their somatic cells to regenerate entire organs or the organism itself. The molecular and cellular events underlying this ability are complex and multifaceted. The initial phase leading to cell cycle reactivation is often called dedifferentiation. This process is triggered either by wounding or exogenous hormone application. In this opinion paper, I propose that the dedifferentiation of mature somatic cells is a two-step process. It involves a transition into a transient senescence-like state induced by stress and/or signals emanating from dying cells. This state entails the loss of genetic information required for cell differentiation, resulting in a critical cellular condition. In the absence of subsequent proliferative signals, dedifferentiating (senescing) cells become committed to programmed cell death. Exogenous and/or endogenous plant hormones, such as auxin and cytokinin, might override this pathway. This rescue step, in most cases, activates cell divisions to replace lost cells/tissues. If cell division is maintained, it may result in callus formation. A callus is not an undifferentiated, homogeneous mass of cells. It is an unorganised tissue with at least some cells having ground-tissue-like molecular identity and high developmental potential. A callus might also form from pre-existing competent cell populations, e.g., pericycle cells, with no senescence-like intermitting state. It is discussed whether this “one-step” callus-formation pathway can be considered dedifferentiation.

## 1. Introduction

The sessile nature of plants necessitates a remarkable capacity for adaptation and regeneration in response to environmental harm and mechanical damage [1,2]. The plant’s regenerative ability, in addition to its meristems, is based on cellular plasticity. Differentiated somatic cells in plants can alter their fate and regenerate a tissue, an organ, or even the entire organism [3,4,5]. The initial process central to this phenomenon is cellular dedifferentiation, a term historically used to describe the reversion of a mature cell to a less specialised state within its own developmental lineage, thereby enabling proliferation [6]. In plant biology, however, there is uncertainty regarding the usage of this term. It is often used broadly to refer to any form of cellular reprogramming that increases developmental potential, regardless of whether it involves a reversal of development [7,8]. An example is the designation of callus as a mass of “dedifferentiated” cells. This may be correct if we consider dedifferentiation as the process leading to callus formation, but it is incorrect to describe the developmental/genetic state of the callus cells [7].

In this paper, I attempt to clarify how dedifferentiation proceeds and how it relates to callus formation. Two primary experimental systems are instrumental in dissecting the mechanisms of plant cell dedifferentiation: the isolation and culture of protoplasts (individual plant cells devoid of their walls) and the induction of callus (an unorganised ground tissue) by wounding or by ectopic hormone levels [9,10,11]. This overview attempts to reconcile observations from these in vitro systems with those of in planta regeneration. I propose that the dedifferentiation of differentiated somatic cells initiates with a senescence-like state, followed by hormonal rescue, leading to proliferation and/or redifferentiation. There is, however, a more direct pathway to callus formation via overproliferation of pre-existing stem or stem cell-like cells. This process does not involve the senescence-like intermediate stage. The applicability of the term dedifferentiation to describe this “one-step” callus formation pathway will be discussed.

## 2. Signals from Dying Cells in the Reprogramming of Differentiated Cells at Wound Sites

The ability to regenerate lost or damaged tissues is a fundamental characteristic of multicellular life. It is essential for survival after injuries and for maintaining organismal integrity. This capability is widespread across the tree of life, yet it differs dramatically among taxa and even within different tissues of the same organism [1]. In the animal kingdom, organisms like planarians and hydra exhibit extraordinary regenerative potential. They can rebuild their entire body from small parts. Mammals, including humans, possess a more limited regeneration ability, primarily restricted to specific tissues such as the liver and skin. Two fundamental, yet seemingly contradictory, processes profoundly contribute to these regeneration strategies: programmed cell death (PCD) and cellular proliferation [12].

Historically, cell death was viewed as a passive and terminal process, the inevitable conclusion of a cell’s life. Over the past few decades, it has become clear that cell death is often an active, genetically controlled program that plays a crucial role in development and tissue homeostasis [13]. More surprisingly, apoptosis, a form of programmed cell death (PCD) of animals, participates in a conserved mechanism for successful tissue repair and regeneration following injury [14,15]. The process is often referred to as “apoptosis-induced proliferation” (AiP). During this process, dying cells release mitogenic signals that stimulate the proliferation of neighbouring stem or progenitor cells to replace lost tissue [12,13]. In mice (*Mus musculus*), for instance, apoptotic cells activate a so-called “phoenix rising” pathway. Key enzymes in the apoptotic cascade, the executioner caspases 3 and 7, trigger the release of growth signals, such as prostaglandin E2, to promote wound healing and liver regeneration [16]. Thus, active communication from dying cells orchestrates the renewal of the collective tissue [12].

In contrast to most animals, plants possess a remarkable capacity for regeneration [17,18]. Many plant species can regenerate entire organs, such as leaves, stems, or roots [19]. In some cases, a whole plant can be reconstituted from a small piece of tissue or even a single cell [4]. Like animals, plants utilise PCD as a fundamental tool for development and for responding to environmental challenges [20]. PCD is essential for sculpting plant architecture, such as during xylem formation. It is also central to some defence strategies, such as the hypersensitive response, in which localised cell death helps contain the spread of pathogens. Given the well-established role of PCD in initiating regeneration in animals, it is logical to hypothesise that a similar principle operates in plants. PCD is deeply integrated into plant defence, but its role as a pro-regenerative signal could create a complex regulatory challenge. The wound response in plants is inherently a balancing act between two potentially conflicting priorities: defence and regeneration [21]. Activating robust defence pathways is energetically costly and often involves growth inhibition, which is antithetical to the cell proliferation required for regeneration. Conversely, prioritising regeneration might leave the plant vulnerable to infection at the wound site. The wound response is a multi-layered process that must simultaneously seal the injury to prevent water loss and pathogen entry and initiate repair and regeneration programs [21,22]. The cells that die at a wound site are not merely casualties of injury; they are the source of instructive signals to activate defence and regeneration. For example, when a cell in the root meristem dies, it loses turgor and collapses. It generates a mechanical signal that leads to elevated auxin accumulation and sensitivity in neighbouring cells, thereby provoking their division [23]. The recognition of damage can also be mediated by the release of molecules from ruptured cells known as Damage-Associated Molecular Patterns (DAMPs) [24]. Wound cues also trigger waves of calcium ions (Ca^2+^), reactive oxygen species (ROS), and electrical signals that propagate from the wound site throughout the plant, alerting even distant tissues to the injury [22]. These early signals are crucial for activating context-dependent local and systemic downstream responses. Local responses often include the PCD of cells at the wound edge [25,26,27]. It is plausible that the genetically controlled dismantling of these cells via PCD could release specific pro-regenerative signals, analogous to the “phoenix rising” pathway in mammals [16]. Indeed, recent work suggests that similar pathways exist in plants. It has previously been shown that when tomato (*Solanum lycopersicum*) plants are damaged, wounding triggers the activation of Ca^2+^-dependent metacaspases, which are also involved in PCD [28]. This leads to the release of peptides that were proposed to bind and activate their extracellular receptors, initiating an immune-like response [29]. These peptides (designated as REGENERATION FACTOR1; REF1) have recently been found to profoundly enhance plant regeneration via receptor-mediated induction of *WOUND-INDUCED DEDIFFERENTIATION 1* (*WIND1*) [30,31], a master regulator of wound-induced cellular reprogramming in plants [32].

The complex network of phytohormones that orchestrate the subsequent cellular and physiological changes is central to the plant wound response. Jasmonic acid (JA), the master regulator of plant defence against herbivores and necrotrophic pathogens, is rapidly synthesised upon wounding [22,33]. JA signalling triggers extensive transcriptional reprogramming, leading to the production of defence compounds and proteins. Besides defence reactions, JA, in concert with ethylene, is a central regulator of PCD and senescence [34,35]. It also triggers the regeneration pathway [36,37,38,39,40] but inhibits subsequent callus formation [41]. In parallel, the phytohormone auxin plays a pivotal role in guiding the regenerative process [5,36,42]. Auxin is a master coordinator of plant development, and its differential accumulation into local maxima is a common mechanism for initiating organ formation. Following wounding, auxin transport is rerouted, and its local synthesis is initiated, leading to its accumulation at the wound site [5,42,43]. There, it triggers cell division, cellular reprogramming, and the formation of new vascular connections or entire organs. An increasing body of evidence also supports a profound and intricate connection between PCD and auxin signalling pathways [44]. This interplay is particularly evident in the context of wound healing and regeneration, where the death of some cells serves as a crucial signal to trigger the restorative division of others. At the heart of this regulatory network lies ETHYLENE RESPONSE FACTOR 115 (ERF115), an AP2/ERF transcription factor. ERF115 has emerged as a pivotal integrator of signals from dying cells and hormonal cues, orchestrating regeneration by activating, among others, *WIND1* expression [26,40].

The function of ERF115 is rooted in its role as a guardian of the stem cell niche [45]. In the *Arabidopsis thaliana* root, ERF115 acts as a rate-limiting factor in the division of quiescent centre (QC) cells, which maintain the surrounding stem cells. Under normal conditions, ERF115 activity is tightly constrained by proteolysis, preventing uncontrolled proliferation and ensuring the longevity of the stem cell niche [46]. This homeostatic balance is disrupted upon injury. Cell death in or near the stem cell niche triggers rapid, localised induction of *ERF115* expression in surviving adjacent cells, which then initiate divisions to replace the lost tissue [3,26]. This principle is observed across various contexts of cellular damage. For instance, wounding of the hypocotyl induces *ERF115* expression within an hour [41]. DNA damage resulting from loss of TOPOISOMERASE1α function triggers PCD in stele stem cells. This cell death is partially compensated for by ERF115-mediated replenishment from neighbouring cells [47]. Similarly, mutations in genes essential for meristem maintenance, such as those coding for MEDIATOR18 and NRPB2, cause spontaneous cell death at the root tip. It invariably correlates with high *ERF115* expression in the surrounding tissue [48]. The close temporal and spatial association between cell death events and *ERF115* induction suggests a form of communication from the dying cell to its neighbours.

The signals emanating from dying cells to activate ERF115 are multifaceted, involving both physical and chemical cues. The physical collapse of damaged cells alters cellular pressure, which neighbouring cells perceive. This spatially defines the regenerative response and ERF115 activation zones [23]. In the unwounded root meristem, *ERF115* expression is limited to the QC. However, as reactive oxygen species (ROS) levels increase, *ERF115* expression extends throughout the entire root meristem [49]. The wound hormone JA was also shown to induce ERF115 transcription, directly linking the plant’s defence and repair pathways [40]. Interestingly, the role of ERF115 is context-dependent. While it typically promotes regeneration, in response to aluminium toxicity, ERF115 participates in a pathway that promotes terminal differentiation and growth arrest following aluminium-induced cell death [50].

While auxin is not the primary inducer of *ERF115* transcription, it is required to maintain high levels of *ERF115* expression during the regeneration process [51]. In turn, ERF115 enhances cells’ sensitivity to auxin, partly by activating the expression of the key auxin response factor *ARF5/MONOPTEROS* (*ARF5/MP*). This mutual amplification ensures a robust and sustained response, granting regenerative competence to otherwise quiescent cells [51]. Auxin’s relationship with PCD is complex; it generally acts as a survival signal that suppresses cell death but can also promote it under certain conditions [44,52,53]. In the context of regeneration, the most critical aspect of this relationship is how PCD alters local auxin dynamics. Dying cells can become a significant source of auxin. For example, the cyclic PCD of lateral root cap cells releases pulses of auxin that pattern the formation of new lateral roots [54]. Furthermore, cell autolysis releases tryptophan from protein breakdown, providing a substrate for local auxin biosynthesis [55]. This suggests that many sites of cell death are also sites of auxin production. Additionally, physical damage and the resulting cell death create an anatomical barrier that obstructs polar auxin transport [51]. In this way, cells adjacent to the wound act like rocks in a stream, leading to auxin accumulation [51]. This localised auxin peak is essential for initiating the regenerative program [23,51,56] and engages in a powerful synergistic feedback loop with ERF115. To execute its function, ERF115 forms a heterodimeric complex with GRAS-domain transcription factors such as PHYTOCHROME A SIGNAL TRANSDUCTION 1 (PAT1) [26,41,57]. This complex then activates a downstream transcriptional cascade, including the DNA-BINDING ONE FINGER 3.4 (DOF3.4) transcription factor, which in turn promotes the expression of D3-type cyclin genes to drive regenerative cell divisions [58].

In summary, while a wound causes necrosis in damaged cells, it also initiates defence and cell death programmes in nearby cell layers. These cells serve as an important source of local and systemic regenerative signals. As such, these cells can reroute themselves and their neighbours for wound closure and/or regeneration. Similar phenomena can be observed in cultured leaf protoplasts. These cells are removed from their tissue environment and are freed from local and systemic hormonal signals (positional information) defining their fate [59].

## 3. The Senescence-like State of Protoplast-Derived Cells

**Figure 1:**
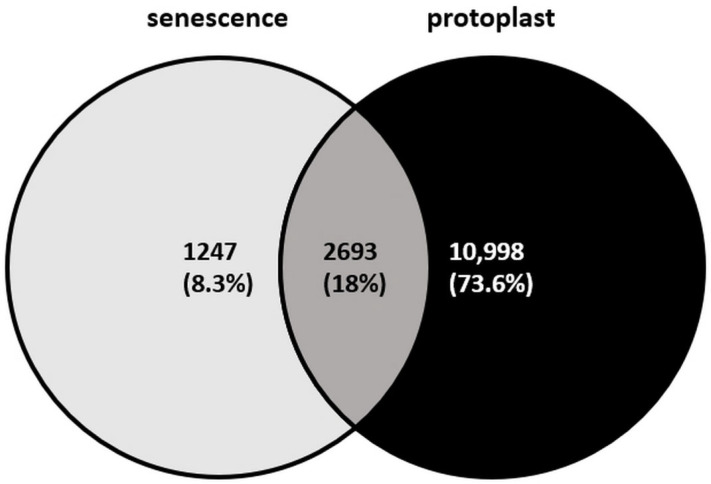
Overlap among the *Arabidopsis thaliana* transcripts associated with leaf senescence [68] and expressed in freshly isolated leaf protoplasts [69].

The isolation of protoplasts from differentiated tissues, such as some of those that form the leaves, provides a powerful system for studying the initial events of cellular reprogramming in a synchronised population of single cells [9,60]. The process itself, involving enzymatic digestion of the cell wall and release into an artificial medium, is a profound stressor. In addition, these cells are removed from their native tissue context and hormonal cues [61,62,63]. The immediate response of protoplasts closely resembles cellular senescence, a programmed developmental process preceding PCD [64,65,66,67]. Cells in both states exhibit similar structural changes. These changes include widespread chromatin decondensation, disruption or shrinkage of the nucleolus, and condensation of ribosomal RNA gene clusters, indicative of reduced protein synthesis and a shift towards a quiescent state [60,64,66]. Furthermore, both processes are marked by a large-scale upregulation of genes from specific transcription factor (TF) families. These include ANAC, WRKY, and bZIP TFs, which are well-known master regulators of stress and senescence responses [60,66]. Consistent with this, transcriptome profiling of dedifferentiating protoplasts reveals a significant overlap with the gene-expression patterns of senescing cells (Figure 1). More than two-thirds of the almost four thousand genes listed in the *Arabidopsis thaliana* senescence database [68] are expressed in freshly isolated leaf protoplasts [69]. These protoplasts transiently but strongly express the *ERF115*, *PAT1*, and *ARF5/MP* transcription factor genes (Table 1, [69]), underscoring their similarity to cells acquiring competence for cell death or wound-induced regeneration [26,45,51,57,70].

The senescence-like transient state was hypothesised to confer pluripotentiality to protoplast-derived cells [71,72]. However, at this stage, the cells can only follow two pathways. If left in a hormone-free medium, they undergo cycles of chromatin condensation and decondensation before ultimately succumbing to programmed cell death [73]. Protoplast-derived cells, which are unable to divide and regenerate even in the presence of plant hormones, are also subjected to programmed cell death [65]. These observations strongly support the idea that the stress-induced, senescence-like state is a direct path to cell death unless the cell is “rescued” by specific proliferative signals that induce endogenous auxin synthesis [74]. The pluripotentiality of proliferating cells is established only under proper culture conditions, including the appropriate ratio of plant hormones [60,69,74]. It has been demonstrated that expression of genes associated with pluripotency, such as *WOX5*, *PLETHORA7* (*PLT7*), and *PLETHORA4*/*BABY BOOM* (*PLT4*/*BBM*), is already detectable in freshly isolated protoplasts, albeit at very low frequency [69]. This random activation was attributed to stochastic changes in chromatin accessibility induced by isolation stress [69]. Expression of pluripotency markers was also observed several days later in proliferating cells during microcallus formation. In addition, Arabidopsis mesophyll cell protoplasts’ regeneration was promoted by the ectopic expression of the *WUSCHEL* (*WUS*) and *DORNRÖSCHEN/ENHANCER OF SHOOT REGENERATION1* (*DRN/ESR1*) pluripotency genes [69].

Seminal work using the tobacco (*Nicotiana tabacum*) protoplast system identified two functionally distinct phases of chromatin decondensation during dedifferentiation and cell cycle reactivation, respectively [22]. The first phase occurs rapidly during protoplast isolation, is independent of exogenous hormones, and corresponds to the loss of differentiated functions, such as photosynthesis, in mesophyll protoplasts. This phase aligns with a stress-induced global chromatin opening, which makes the genome accessible to the senescence-associated transcriptional program [71]. This initial reprogramming involves marked changes in the expression of chromatin-associated genes, particularly those encoding histone variants, and is associated with epigenetic modifications [60,71,72,75,76]. Once a cell has entered the plastic, senescence-like state, its fate is largely dictated by the hormonal milieu. In this context, the application of a protoplast culture medium typically rich in auxin and supplemented with cytokinin serves as a critical rescue signal [73,77]. However, recent studies have shown that exogenous auxin alone is insufficient and that endogenous auxin synthesis is required for the initial cell division of protoplast-derived cells [74]. The second phase of chromatin decondensation occurs only after protoplasts are supplied with phytohormones (in most cases, with auxin and cytokinin) and precedes endogenous auxin synthesis and the cell’s entry into S-phase [71,74,75,78]. The competent cells complete the mitotic cycle, thereby averting cell death and initiating callus formation [60,69,71,74].

Callus formation, however, can be dissected from the senescence-like dedifferentiation process. The maintenance of the differentiated state in mature plant cells is an active process, partly enforced by epigenetic silencing mechanisms. For example, the POLYCOMB REPRESSIVE COMPLEX 2 (PRC2), which deposits repressive histone marks, plays a pivotal role in preventing the unscheduled dedifferentiation of mature somatic cells like root hairs. Loss of PRC2 function can lead to spontaneous reprogramming of single differentiated cells. However, since these cells remain under hormonal influence, they can directly regain cell division activity, leading to callus formation or even somatic embryogenesis [79]. In contrast, reactivation of cell division in mesophyll cells requires histone deacetylation, and inhibiting this process prevents callus formation [74,80].

Therefore, I propose that the dedifferentiation of mesophyll protoplast-derived cells is a two-phase process: it begins with a senescence-like event that leads to the loss of differentiated functions, followed by cell proliferation and redifferentiation or callus formation. However, can we consider this two-step process as dedifferentiation in a developmental biology sense? To do so, we must accept that the callus cells are the developmental predecessors of mesophyll cells.

## 4. The Formation and Nature of Callus Tissues

For decades, callus was described as a mass of “undifferentiated” or “dedifferentiated” cells. This view is increasingly being challenged by molecular data suggesting that callus is not a developmental void but rather a specific tissue state with a characteristic gene expression signature [7,11,41,81]. A comprehensive comparison of transcriptomic datasets from Arabidopsis calli of various origins and ages has identified a core set of 21 genes that are consistently upregulated and may define this state [11].

If one accepts this gene set as a relevant molecular signature, it is highly informative of the nature of the callus tissue. It includes key transcription factors that are central to embryonic and post-embryonic development, particularly in the formation and maintenance of meristems and organ primordia [11]. Among these are ARF5/MP and AINTEGUMENTA-LIKE 6/PLETHORA 3 (AIL6/PLT3), which are important regulators controlling ground, vascular, and stem cell identities, meristem formation, maintenance, and function, as well as the initiation and development of lateral organ primordia (e.g., [82,83,84,85,86,87,88,89,90]). Other core callus genes include the genes of the TFs SPATULA (SPT) and WRKY23, both of which regulate meristem size and auxin-dependent developmental processes [91,92,93,94]. The consistent expression of these and other meristem- and provascular-associated genes [11,81] suggests that calli, at least in certain regions [81], represent a state characteristic of a developmentally plastic (embryonic, meristematic, primordial, provascular) ground tissue. In in vitro culture, this state is prevented from progressing to organised development, such as embryogenesis or organogenesis, by exogenous hormones. Instead, the hormonal conditions in the culture medium cause overproliferation. The same may hold for wound-induced in planta callus formation depending on hormonal gradients and sensitivities of the damaged tissues/organs. The embryonic/meristematic/primordial ground tissue nature of calli explains their high regenerative potential. They contain cell populations that can be readily induced to form new meristems and organs upon transfer to appropriate regeneration media.

If we accept that calli resemble the embryonic/meristematic ground tissue from which internal tissues differentiate, then callus formation from these tissues can be considered de facto dedifferentiation in the developmental biology sense (i.e., stepping back in the developmental lineage). Interestingly, organ or embryo regeneration from epidermal tissues, often from the base cells of glandular trichomes, proceeds directly without callus formation [95,96,97]. However, epidermal protoplasts can also divide and form cell colonies [98]. Since epidermal cells are not of ground tissue origin, this might not be considered as dedifferentiation in the classical sense, except that the callus represents a very early, pre-meristematic, or even embryonic stage [99]. Another explanation is that, under in vitro culture conditions, cell differentiation cannot revert from protoplastation-induced senescence, and thus the cells acquire a new fate. It means they would rather transdifferentiate than dedifferentiate into a callus. Extensive chromatin remodelling and stochastic gene expression induced by stress and exogenous hormones may facilitate this developmental shift [69].

The dramatic reprogramming of terminally differentiated cells via a senescence-like intermediate is not the only route to callus formation. A substantial body of evidence indicates that, particularly in response to exogenous auxin, callus can arise from specific, pre-existing populations of competent cells [8,10,17,100,101].

In Arabidopsis, auxin-induced callus from various explants, including roots and aerial organs, has been shown to originate from pericycle or pericycle-like cells [100,101,102]. This process hijacks the developmental program for lateral root formation. The signalling cascade involves the auxin-mediated degradation of repressor proteins, leading to the activation of ARF7 and ARF19, which in turn induce the expression of genes coding for LATERAL ORGAN BOUNDARY (LBD) transcription factors [10,41,102] leading to the emergence of lateral root primordia. In this scenario, the callus can be viewed as an over-proliferating, disorganised lateral root primordium [7,11]. It forms from cells that are already endowed with cell division competence and a degree of developmental plasticity [8,101,103,104]. Therefore, in my view, auxin-induced callus formation in intact tissues that do not involve differentiated somatic cells might not constitute a classic example of dedifferentiation.

## 5. Discussion

In this opinion paper, I propose that plant cell dedifferentiation is context-dependent; thus, the term “dedifferentiation” does not always have a uniform meaning. Somatic plant cells have different competences to re-enter the cell division cycle and regenerate lost cells or tissues. This competence is defined, among other factors, by long-distance hormonal gradients that depend on the distance of cells from the meristems [43,51,105]. Moreover, certain cells, such as pericycle or pericycle-like cells, have been shown to maintain this competence, even after leaving the meristematic region during growth [103,104].

In my view, somatic cells, which lack inherent competence for a fate switch, follow a two-step dedifferentiation pathway. Initially, stress/wound induces entry into a senescence-like transient state (Figure 1). This is followed by a second phase: a hormonal rescue into a proliferative state. This two-step pathway underlies the mechanism required to reprogram, for example, leaf mesophyll protoplasts that lack inherent plasticity [71]. The protoplast isolation-induced initial senescence-like phase effectively erases the differentiated state at the chromatin and gene expression levels. This creates a blank slate upon which a new developmental program can be written. However, in the absence of subsequent proliferation and/or differentiation signals from exogenous and/or endogenous hormones, this state is a dead end, leading to cellular demise [71].

It has been proposed that dedifferentiating leaf protoplast-derived cells are in a pluripotent stem cell-like state [71,72,75]. In this hypothesis, the term ‘pluripotency’ was used in a very broad sense. Three potential developmental trajectories were considered, leading towards death, redifferentiation or cell division. According to this scenario, dedifferentiation precedes the reactivation of cell division, which is only one of the possible outcomes. Recent research indicated that pluripotency regulators of the shoot meristem (such as WUS and DRN) are stochastically activated already in freshly isolated leaf protoplasts and promote their regeneration [69]. It remains, however, a question whether mesophyll protoplast-derived cells are formed by dedifferentiation if their gene expression patterns are stochastic [69,75]. Dedifferentiation of mesophyll cells should theoretically result in cells resembling the cells from which they differentiate: the ground tissue in young leaf primordia and/or in the shoot meristem. There are indications that callus shares some gene-expression characteristics with ground tissues of young embryos, meristems, and organ primordia [11]. The expression of these marker genes is also induced in protoplast-derived microcalli [11]. Therefore, dedifferentiation (here, the acquisition of ground tissue characteristics) may occur during the proliferative stage of protoplast cultures. One possible scenario is that the transient senescence-like state not only erases differentiated genetic information but also results in the stochastic, sporadic activation of pluripotency factors [69]; however, the actual cell-fate switch occurs upon exogenous hormone application. The application of auxin alone results in the differentiation of elongated parenchymatic cells, while, together with cytokinin, it induces and maintains cell divisions [73]. Without hormonal gradients controlling tissue patterning, additional cell proliferation results in disorganised callus growth.

In whole plants, cell replacement and/or wound healing also require the reactivation of cell division in cell death- or wound-adjacent cells. In this case, it is more difficult to dissect the relationship between cell division and dedifferentiation. Cells in the meristematic region have been shown to regain stem cell abilities prior to restorative cell division [37,106]. The regulatory steps that directly activate stem cell identity factors in these cells remain unknown [37]; however, auxin accumulation is a strong candidate for mediating this activation [51]. The regenerative ability of cells is closely associated with the expression of several AP2/ERF transcription factor genes, including *ERF109*, *ERF115*, and *WIND1*, which regulate local hormone synthesis and sensitivity [36]. These factors have been shown to be activated by various signals from dying cells [23,30,31,51]. I propose that these signals induce a similar transient senescence-like state in wound-adjacent cells, as observed following protoplast isolation. This idea is supported, for example, by the expression of the *ERF115* regeneration factor gene in both freshly isolated protoplasts and wounded tissues (Table 1).

If cell damage is extensive and distant from the meristems, the wound-induced ERF115-WIND1 pathway leads to callus formation [10,32,36,37]. In these scenarios, cell divisions may also be triggered, leading to wound closure by callus formation, but organ formation cannot occur without appropriate patterning information.

An alternative pathway of callus formation involves the direct hormone-mediated activation of pre-existing competent cells, such as those of the pericycle [10]. These cells, by their nature and position, retain a higher degree of developmental potential and may not need to undergo the drastic and risky process of complete reprogramming via a senescence-like intermediate. Instead, they can be directly co-opted into a new developmental trajectory, such as lateral root formation [102]. This pathway, however, can be diverted into less organised callus growth under the influence of ectopic exogenous and/or endogenous hormones, especially auxin and cytokinin [98,99]. It is a question whether lateral root primordia formation from pericycle cells involves a dedifferentiation step. Since pericycle cells originate from the root meristem, the formation of lateral root primordia from these cells might be considered a step back in the developmental lineage and thus a form of dedifferentiation. However, it is certainly not the case when the lateral root initiation pathway is induced in aerial organs [8,101]. The next step, callus formation from the emerging root primordia in the continuous presence and uniform distribution of cell division-activating plant hormones in the medium, in my view, is uncontrolled growth. Nevertheless, the developing callus maintains the expression of meristematic genes and thus a high developmental potential.

In summary, two main modes of reprogramming leading to regeneration or callus formation are proposed. The first involves a two-step process where severe stress causes a terminally differentiated cell to enter a temporary, senescence-like state of competence. It can be rescued from further senescence and eventual death, returning to a proliferative state through the induction of regeneration factors and hormonal signals. This process can be considered de facto dedifferentiation, namely, a step back into either a ground cell (callus formation) or a stem cell-like (direct regeneration) state. It is followed either by cell, tissue, or organ replacement when proper hormonal gradients are present or by callus formation if patterning information is overridden by ectopic hormone levels. The second is a more direct pathway in which a new organ or callus is initiated by pre-existing competent cells that are already poised for division and development. The outcome of this pathway, organ or callus, also depends on hormonal cues. This pathway, in my view, does not fall into the classic definition of dedifferentiation. Thus, in my view, callus can develop via either dedifferentiation or disorganisation. In both cases, callus formation follows the initial stages of organ regeneration or development, as these are the default pathways. It proceeds, however, with uncontrolled proliferation under exo- or endogenous hormonal influence, preventing tissue organisation.

## 6. Conclusions

The dedifferentiation of plant cells is considered a cornerstone of their developmental plasticity and regenerative capacity. It is a context-dependent process with several layers that depend on parameters such as the initial cell type, its position in the organism, and the induction conditions. Therefore, it is difficult to establish a clear-cut definition of it. The classical definition, as a return to earlier stages within the developmental lineage, is difficult to apply universally in plant science. One probable reason is that cell lineage is a less important factor in plant cell differentiation than positional information defined by hormonal gradients and cell-to-cell communication. The classical definition seems to apply to regenerating meristems. Our understanding of the process in other, “more differentiated”, tissues is, however, rather limited. One common theme of wound-induced dedifferentiation appears to be the activation of regeneration pathways, involving the ERF115/WIND1 and related regeneration factors. However, these pathways are non-specific, as they are also involved in other developmental processes, such as lateral root development and programmed cell death, which also involve mechanical signals. Thus, activation of this pathway is insufficient to define dedifferentiation. The regeneration factors alter hormone levels and sensitivities, thereby inducing cell divisions. However, the subsequent steps are position-dependent and may result in organised cell/tissue/organ replacement or unorganised callus formation. This second possibility occurs during hormonally induced callus formation of lateral root primordia. The unorganised growth of organ primordia is often called dedifferentiation in the scientific literature, even including the initiation of the primordia from the pre-destined competent cells.

Thus, in plant cell biology, the term “dedifferentiation” is used to describe multiple processes. These processes do not always depend on dedifferentiation in the traditional sense, as used in animal developmental biology. However, are all connected to regeneration or organ formation; nevertheless, under certain conditions, the underlying developmental pathway is diverted towards disorganised callus formation. Many unresolved questions remain regarding the various mechanisms of plant cell dedifferentiation. How specific or widespread are the regenerative signals from dying cells, particularly in light of the recently identified REF1 peptide? Is the cell differentiation-specific information erased in a stochastic or in a controlled way during dedifferentiation? Does this erasing proceed similarly during dedifferentiation and senescence? How much do the two processes overlap, especially as chromatin remodelling is considered? Do calli indeed represent an early stage of plant/organ development? At what stage do normal organ development and organ regeneration converge to the same regulatory mechanism? How do auxin and cytokinin prevent this during callus formation? It is necessary to track cell-fate switches across diverse cell types and systems to gain a more comprehensive understanding of the underlying processes.

Recognising that the term “dedifferentiation” may encompass distinct biological processes is essential to advance our fundamental understanding of plant regeneration. This view, in addition to clarifying long-standing debates over terminology, might also help improve plant tissue culture and biotechnological applications, which depend on the efficient and predictable manipulation of plant cell fate.

## Figures and Tables

**Table 1:** Differential expression of three regeneration-related transcription factors in freshly isolated and dividing 4-day-old *Arabidopsis thaliana* leaf protoplasts from the transcriptomic analysis of Xu et al. [69].

		Protoplasts Versus Leaf Cells			4-Day-Old Versus 0-Day-Old Protoplasts		
	GID	log_2_FC	*p* Value	FDR	log_2_FC	*p* Value	FDR
ERF115	AT5G07310	11.45	1.8251 × 10^−37^	0.00	−1.31	0.00135717	0.00205361
PAT1	AT5G48150	0.75	8.1593 × 10^−6^	1.3381 × 10^−5^	−2.18	3.7995 × 10^−35^	4.3449 × 10^−34^
ARF5	AT1G19850	6.59	1.169 × 10^−142^	2.779 × 10^−140^	−0.79	2.7827 × 10^−5^	4.9201 × 10^−5^

log_2_FC—log_2_ value of fold change; *p* value—level of significance; FDR—false discovery rate.

## Data Availability

No new data were created or analysed in this study.
